# Proteomics Profiling Reveals Pharmaceutical Excipient PEG400 Induces Nuclear-Receptor-Activation-Affected Lipid Metabolism and Metabolic Enzyme Expression

**DOI:** 10.3390/ijms26041732

**Published:** 2025-02-18

**Authors:** Mei Zhao, Siyuan Cao, Dan Yang, Leyuan Shang, Ye Hang, Pengjiao Wang, Shuo Zhang, Chaoji Li, Min Zhang, Xiuli Gao

**Affiliations:** 1 School of Basic Medicine & State Key Laboratory of Discovery and Utilization of Functional Components in Traditional Chinese Medicine & School of Pharmaceutical Sciences, Guizhou Medical University, Guiyang 561113, China; 2 Microbiology and Biochemical Pharmaceutical Engineering Center of Guizhou Provincial Department of Education, Guizhou Medical University, Guiyang 561113, China; 3 Experimental Animal Center, Guizhou Medical University, Guiyang 561113, China

**Keywords:** PEG400, proteomic, retinol metabolism, bile secretion, nuclear receptor, metabolic enzymes

## Abstract

PEG400 is widely used as a pharmaceutical excipient in the biomedical field. Increasing evidence suggests that PEG400 is not an inert drug carrier; it can influence the activity of various drug-metabolizing enzymes and transporters, thereby affecting the in vivo process of drugs. It can also alleviate obesity and adipose tissue inflammation induced by a high-fat diet. In this study, we employed proteomics to investigate the impact of PEG400 on hepatic protein expression in rats. We found that over 40 metabolic enzymes were altered, with UDP-glucuronosyltransferase 1a9 (Ugt1a9) showing the most significant upregulation. This observation is consistent with our previous findings. KEGG pathway enrichment analysis revealed that PEG400 influences retinol metabolism, steroid hormone biosynthesis, drug metabolism, bile secretion, fatty acid degradation, peroxisome proliferator-activated receptor (PPAR) signaling pathway, and pentose and glucuronate interconversions. Western blot and molecular docking were used to quantitatively analyze related proteins. The results demonstrated that PEG400 promotes the metabolism of retinol to produce retinoic acid; enhances bile secretion by upregulating bile acid synthesis and transporter proteins; and activates the PPARα signaling pathway to regulate the expression of fat metabolism-related proteins, thereby reducing lipid accumulation. Furthermore, as natural ligands for nuclear receptors, retinoic acid and bile acids may activate nuclear receptors and initiate the regulation of target gene expression. We found upregulation of the nuclear receptors PPARα, retinoid X receptor alpha (RXRα), and pregnane X receptor (PXR). RXRα can form a dimer with PPARα or PXR to regulate the expression of target genes, which may explain the changes in the expression of numerous metabolic enzymes. This study provides a comprehensive understanding of the effects of PEG400 on liver metabolism in rats, reveals its potential biological functions, and offers new insights into the application and development of PEG400.

## 1. Introduction

Pharmaceutical excipients refer to the additives and substances used in drug production and prescription formulation, which are essential components of drugs. In a broad sense, pharmaceutical excipients are considered necessary ingredients in the formulation of pharmaceutical preparations, in addition to the active drug ingredients. They determine the drug’s dosage form and enable it to exert its optimal curative effect [1]. Pharmaceutical excipients have traditionally been assumed to be pharmacologically inert and not to affect the pharmacological action, content determination, or stability of the drugs in pharmaceutical formulations. However, numerous studies have shown that pharmaceutical excipients can regulate the transportation and metabolism of drugs in the body [2].

The pharmaceutical excipient polyethylene glycol 400 (PEG400) is a product of the polycondensation of ethylene oxide and water, and it exhibits excellent water solubility and biocompatibility. Due to its unique properties, PEG400 is widely used in soft capsules, injections, liposomes, and other pharmaceutical preparations. As a low molecular weight polymer, PEG400 can be taken up by cells through passive transport and interact with intracellular proteins [3]. Studies have shown that PEG400 has a clear influence on the drug-metabolizing enzyme cytochrome oxidase (CYP) and UDP-glucuronosyltransferase (UGT), impacting the phase I and II metabolic process of drugs [4]. PEG400 has an inhibitory effect on the activity of drug transport proteins [5]. Some experiments have also shown that PEG400 can regulate the expression of mRNA of the rat gastrointestinal efflux transporter [6]. Moreover, Studies have demonstrated that oral administration of 40% PEG400 can modify the composition of gut microbiota, significantly increase the abundance of *Akkermansia muciniphila* (*Akk*) bacteria, and alleviate obesity induced by a high-fat diet, this study uncovers more potential clinical applications of PEG 400 from the perspective of disease treatment [7]. We also earlier found the effect of PEG400 on lipid metabolism in mice. High-dose PEG400 significantly increased the abundance of *Akk* bacteria in the gut microbiota of mice, resulting in alterations to the fecal metabolome and impacting lipid metabolism and energy metabolism in mice [8].

Our previous studies found that PEG400 significantly increases the absorption of baicalin and its metabolites in the body and the tissue distribution process; it also increases the retention of baicalin and its metabolites in cells [9,10,11,12]. The mechanism may be realized by modulation of phase II metabolic enzymes and drug transporters. We further demonstrated that PEG400 elicited a significant increase in the enzymatic activity of Ugt1a8 and Ugt1a9, while concurrently inhibiting the activity of efflux transporters in vitro [10,11,12]. The nuclear receptor family has been shown to regulate the expression of metabolic enzymes and transporters. It remains to be investigated whether the effect of PEG400 on metabolic enzymes and transporters is related to the activation of nuclear receptors. A recent study has shown that PEG400 was the optimal linker for the PEGylated DC-targeted mRNA vaccines; as a drug modification material, PEG400 can be efficiently taken up by dendritic cells, thereby effectively inhibiting the growth of tumor cells and prolong the survival time of tumor-bearing mice [13]. Therefore, it is worth additional consideration to consider the in vivo effects of PEG400 on the drug process and its biological effects. Currently, there is a lack of research on the impact of pharmaceutical excipients during drug therapy. Whether PEG400 is used as a pharmaceutical excipient or a drug modification material, its functional effects in vivo should be paid attention to.

Protein is a biological effector, a direct manifestation of life activity, and changes in protein levels can be an indication of changes in physiological function. Proteomics technology enables the systematic identification and quantification of all proteins present in cells, tissues, or organisms. Through screening different proteins, the target of the drug’s action on the body can be found and the effect on the body is accurately reflected [14,15]. Proteomics research can unveil the molecular mechanisms underlying specific biological processes and diseases, and it has been widely employed in basic research, clinical diagnosis, and drug development.

In this study, proteomics was applied to comprehensively analyze the effect of PEG400 on protein expression in rat liver. Differentially expressed proteins were screened out, and bioinformatics methods such as Gene Ontology (GO) enrichment, Kyoto Encyclopedia of Genes and Genomes (KEGG) functional analysis, and Protein–protein Interaction (PPI) were employed to identify significant alterations in signaling pathways. The differential proteins were analyzed by Western blot (WB), enzyme-linked immunosorbent assay (ELISA), and molecular docking to elucidate the scientific significance of the effect of PEG400 on rat liver metabolism. This study aims to provide additional experimental data on the effect of PEG400 on drug metabolism to provide more data support for the clinical application of PEG400 and the development of new formulations.

## 2. Results

### 2.1. Histomorphology and Body Weight of Rat Liver

**Figure 1:**
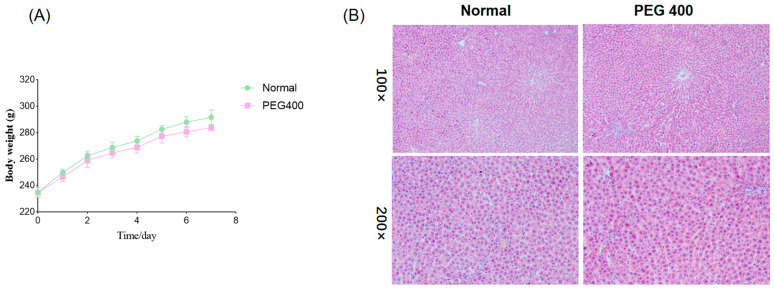
Effect of PEG400 on the general condition of rats. (**A**) Changes in body weight. (**B**) HE staining of the liver.

During the PEG400 oral administration period, the rats exhibited good general health with no signs of diarrhea or loose stools, and their food and water intake showed no differences compared to the control group. As shown in Figure 1A, body weight gain in the PEG400 group was slower compared to the control group. Figure 1B demonstrates that there were no significant differences in liver histomorphology between the control and PEG400 groups. The liver tissue in both groups appeared normal, with clear and intact hepatic lobules, portal areas, and other structures. Hepatocytes were polygonal, well-arranged, and displayed abundant cytoplasm, large and round nuclei centrally located. These findings indicate that PEG400 had no adverse effects on the liver tissue morphology of the rats.

### 2.2. Proteomic Profiling Results

#### 2.2.1. Differential Protein Screening and Analysis

**Figure 2:**
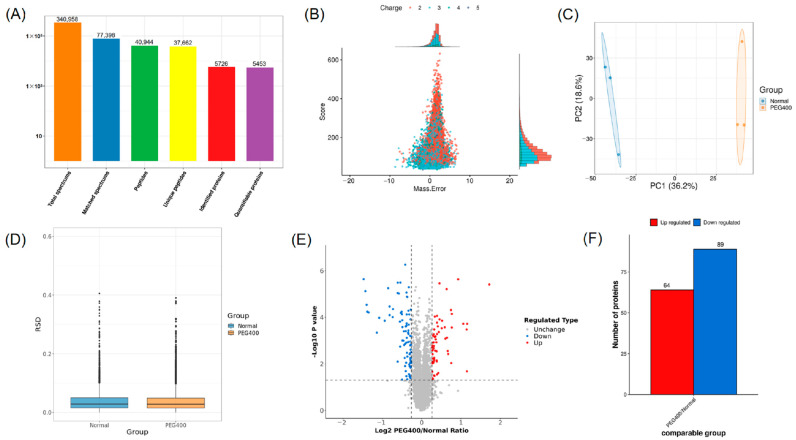
Screening and analysis of differential proteins by proteomics. (**A**) The summary of MS identification results. (**B**) The distribution map of peptide mass tolerance. (**C**) Results of principal component analysis. (**D**) Comparison of relative standard deviation. (**E**) Volcano plot of proteomic analysis. (**F**) Bar graph of differential protein analysis.

To comprehensively elucidate the effects of PEG400 on the rat liver proteome, we employed TMT-based quantitative proteomics to analyze liver tissue samples from the control and PEG400 groups. A total of 340,958 spectra were obtained, leading to the identification of 5726 proteins, of which 5453 proteins were accurately quantified (Figure 2A). According to the peptide mass tolerance distribution (Figure 2B), the majority of spectra had a primary mass error within 10 ppm, meeting quality control standards. Principal component analysis (PCA) and relative standard deviation (RSD) were used to assess the reproducibility of protein quantification (Figure 2C,D), and the results showed excellent reproducibility among the samples. The degree of differential protein expression between groups was analyzed using volcano plots and bar charts. As shown in Figure 2E,F, compared to the control group, 64 proteins were upregulated and 89 proteins were downregulated in the PEG400 group. These results indicate that PEG400 induced changes in the protein expression profile of the rat liver. Notably, more than 40 metabolic enzymes showed altered expression in the PEG400 group compared to the control group. These enzymes include CYPs, flavin-containing monooxygenases (Fmo), alcohol dehydrogenases (Adhs), and aldehyde dehydrogenases (Aldhs), as well as phase II metabolic enzymes such as UDP-glucuronosyltransferases (Ugts).

#### 2.2.2. GO and KEGG Enrichment Analysis

**Figure 3:**
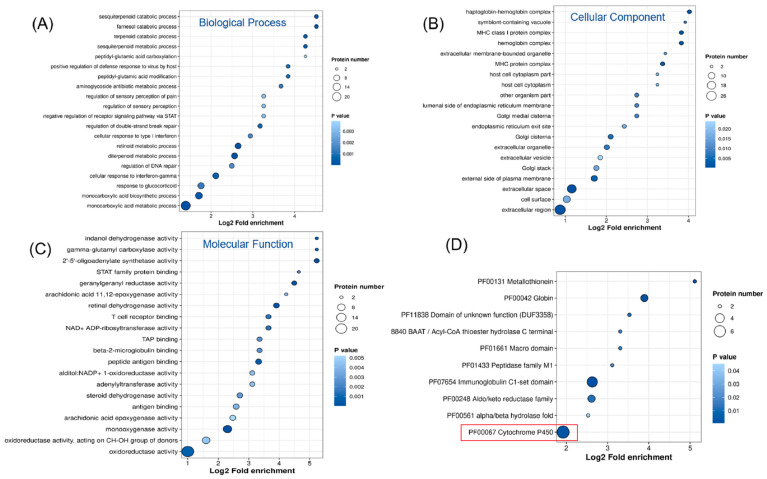
GO enrichment analysis for proteomics. (**A**–**C**) Bubble plot of the effect of PEG400 on GO enrichment analysis of biological processes, cellular components, and molecular functions. (**D**) Enrichment analysis of PEG400 effects on protein domains.

**Figure 4:**
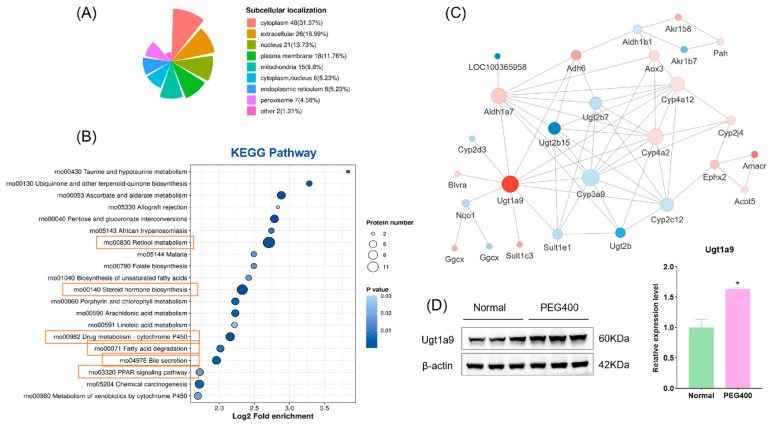
KEGG pathway enrichment analysis of the effect of PEG400 on liver proteomics. (**A**) Subcellular localization analysis. (**B**) KEGG pathway enrichment analysis. (**C**) Protein interaction network analysis. (**D**) WB detection and quantitative analysis of Ugt1a9 protein expression. * *p* < 0.05.

Differentially expressed proteins were subjected to enrichment analysis using the GO database, which includes three categories: biological processes (BPs), cellular components (CCs), and molecular functions (MFs). As shown in Figure 3A–C, the GO enrichment analysis revealed significant changes across all three categories. Regarding biological processes, the differentially expressed proteins were primarily enriched in monocarboxylic acid biosynthesis and transport, retinol metabolism, diterpenoid metabolism, and cellular responses. The proteins were mainly enriched in the cell membrane and extracellular matrix for cellular components. The most significant enrichment in molecular functions occurred in oxidoreductase, dehydrogenase, and transferase activities. To further elucidate the functions of these differentially expressed proteins, we conducted protein domain enrichment analysis, which revealed that CYP450 enzymes were the most significantly enriched (Figure 3D). This finding indicates that PEG400 can impact drug metabolism. Subcellular localization analysis (Figure 4A) showed that most differentially expressed proteins were located in the cytoplasm (31%), followed by the extracellular matrix (17%) and nucleus (14%). A smaller proportion of proteins were localized in the cell membrane (12%) and mitochondria (10%). Notably, we observed that the number of downregulated proteins in the cell membrane (14 proteins) was significantly higher than that of upregulated proteins (4 proteins). This suggests that PEG400 may predominantly suppress the expression of membrane transport proteins. This observation aligns with previous literature on the effects of PEG400 on efflux transporters [5,6,12]. KEGG pathway enrichment analysis (Figure 4B) revealed that retinol metabolism and steroid hormone biosynthesis were the most significantly enriched pathways, followed by CYP450-mediated drug metabolism, bile secretion, PPAR signaling, pentose and glucuronate interconversion, ascorbate and urate metabolism, and fatty acid degradation.

#### 2.2.3. PPI Network Analysis

The differentially expressed proteins were further analyzed using PPI network analysis, resulting in a complex interaction network containing 27 nodes and 63 edges (Figure 4C). In the network diagram, circles represent the differentially expressed proteins, with colors indicating the degree of expression changes (blue for downregulated proteins and red for upregulated proteins). The color intensity corresponds to the magnitude of fold change. Among the proteins, the most significantly upregulated was Ugt1a9, a phase II metabolic enzyme, with a fold change of 3.3 times that of the control group. Western blot analysis confirmed significant upregulation of Ugt1a9 protein expression in the PEG400 group (Figure 4D). Other key nodes in the network included Cyp4a2, Cyp4a12, Cyp3a9, Aldh1a7, Ugt2b7, and Ugt2b15. The results indicate that the differentially expressed proteins are interconnected and interact, forming the molecular basis of PEG400’s effects on rat liver metabolism.

### 2.3. Effect of PEG400 on Retinol Metabolism

**Figure 5:**
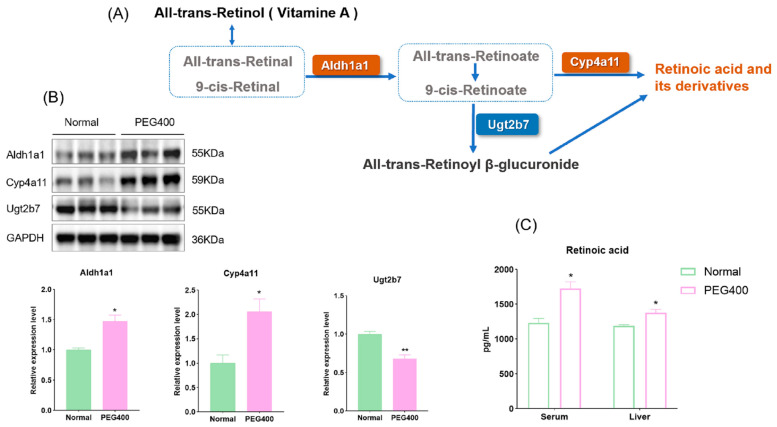
Effect of PEG400 on retinol metabolism. (**A**) Possible schematic representation of retinol metabolism to retinoic acid. (**B**) WB detection and quantification of the expression of enzymes involved in retinol metabolism. (**C**) Measurement of retinoic acid content in liver and serum. * *p* < 0.05, ** *p* < 0.01.

KEGG pathway enrichment analysis revealed that retinol metabolism was the most significantly affected metabolic pathway by PEG400 in rat liver. We used WB to quantify the expression of enzymes related to retinol metabolism among the differentially expressed proteins. As shown in the retinol metabolic pathway diagram (Figure 5A,B), PEG400 promotes retinol conversion to RA by upregulating the protein expression of Aldh1a1 and Cyp4a11. Simultaneously, PEG400 downregulates the expression of Ugt2b7, reducing the glucuronidation-mediated inactivation of RA. We further measured RA levels in serum and liver samples. As shown in Figure 5C, the levels of RA in both liver and serum were significantly higher in the PEG400 group compared to the control group. RA is an active metabolite of retinol, functioning as a lipophilic signaling molecule and representing the primary physiological form of retinol activity in vivo. These results indicate that PEG400 promotes retinol metabolism by influencing the expression of metabolic enzymes.

### 2.4. PEG400 Promotes Bile Secretion

**Figure 6:**
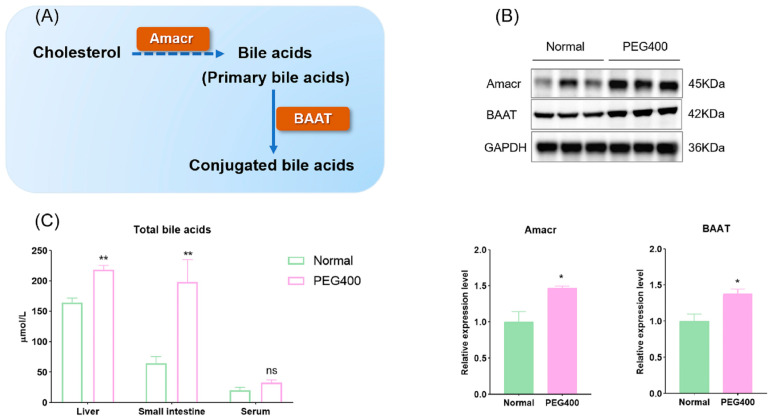
Effect of PEG400 on bile acid synthesis. (**A**) Schematic diagram of the production of bile acids. (**B**) Expression and quantification of Amacr and BAAT by WB. (**C**) Determination of total bile acid content in serum and liver of rats. * *p* < 0.05, ** *p* < 0.01, ns *p* > 0.05.

KEGG pathway analysis also indicated that PEG400 affects bile secretion. Proteomic analysis showed that the expression of Alpha-methylacyl-CoA racemase (Amacr) was upregulated by 2.2-fold in the PEG400 group compared to the control group. To validate this finding, we performed Western blot analysis to quantify the expression of Amacr and bile acid-CoA: amino acid N-acyltransferase (BAAT), another enzyme critical for bile acid synthesis. Both proteins were significantly upregulated in the PEG400 group compared to the control group (Figure 6A,B). We also measured the total bile acid content in serum, liver, and small intestine using commercial assay kits. The results showed that total bile acid levels were significantly elevated in the liver and small intestine of the PEG400 group, while no significant changes were observed in serum bile acid levels (Figure 6C). These findings suggest that PEG400 enhances bile acid synthesis and transport processes in the liver and intestine.

### 2.5. PEG400 Affects the Activation of the PPAR Signaling Pathway

**Figure 7:**
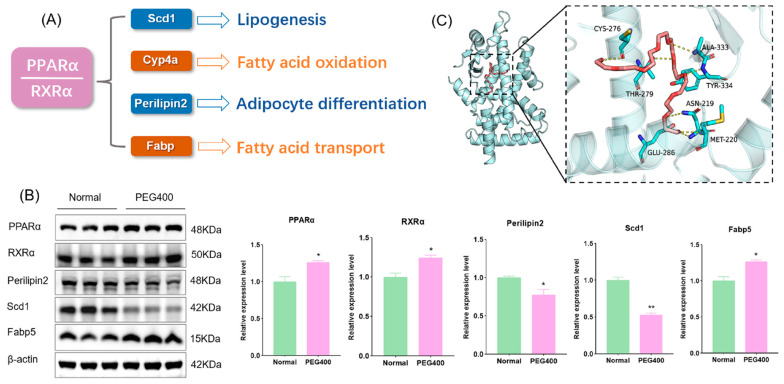
Effect of PEG400 on PPARα signaling pathway. (**A**) PPARα signaling pathway regulates fatty acid metabolism. (**B**) WB detection and quantitative analysis of PPARα signaling pathway-related protein expression. (**C**) Molecular model of PEG400 binding to PPARα protein. * *p* < 0.05, ** *p* < 0.01. The cyan cartoon indicates the PPARα protein, the long stick of the red series indicate PEG400 molecules, and the short stick of the blue-colored series indicate amino acid residues of the protein, the yellow dashed line indicates hydrogen bonding.

PEG400 influences the PPAR signaling pathway and fatty acid degradation. Peroxisome proliferator-activated receptors (PPARs) play a critical role in regulating lipid metabolism and energy homeostasis. PPARs include three subtypes: PPARα, PPARβ/δ, and PPARγ, among which PPARα primarily regulates fatty acid oxidation in the liver. PPAR binds to the retinoid X receptor (RXR) to form a heterodimer that regulates the expression of target genes. We used WB to detect the expression of PPARα, RXRα, and its downstream target proteins involved in fatty acid metabolism. The results revealed significant changes in the expression of PPARα-related proteins in the PEG400 group, including fatty acid-binding protein (Fabp), which facilitates fatty acid transport, stearoyl-CoA desaturase (Scd1), and perilipin 2, both of which regulate adipocyte formation and differentiation (Figure 7A,B). Scd1, a central lipogenic enzyme, is the final rate-limiting step of glucose and lipid metabolism and is a potential target for controlling lipogenesis [16]. Fabp is a family of proteins involved in fatty acid transport. Fabp plays a crucial role in fatty acid transport, nuclear signal transduction, and lipid metabolism. As a target in lipid metabolism research, Fabp5 can bind to fatty acid ligands, further activating nuclear receptor PPARα and regulating fatty acid metabolism [17]. Perilipin 2 is involved in the regulation of lipid metabolism and assists in lipid storage [18]. These findings indicate that PEG400 induces activation of the PPARα signaling pathway. Upon activation, PPARα forms a heterodimer with RXRα and initiates adipocyte signaling pathways. This activation downregulates the expression of Scd1 and perilipin 2, reducing adipocyte formation and differentiation, while upregulating Cyp4a and Fabp5 to promote fatty acid β-oxidation, thereby reducing lipid accumulation [19,20,21]. Additionally, molecular docking analysis was performed to examine the interaction between PEG400 and PPARα. The results showed that PEG400 could effectively bind to PPARα, with a binding energy of −6.3 KJ/mol (Figure 7C). This suggests that PEG400, as a lipophilic molecule, may directly activate PPARα. The activation of RXRα may be related to the increase in its natural ligand retinoic acid.

### 2.6. Effect of PEG400 on Metabolic Enzymes

**Figure 8:**
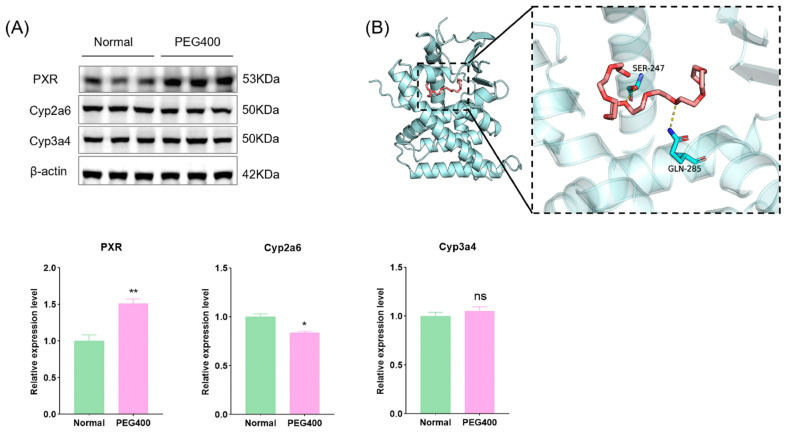
Effect of PEG400 on nuclear receptor PXR and PXR-regulated CYP enzymes. (**A**) WB detection and quantitative analysis. (**B**) Molecular model of PEG400 binding to PXR. * *p* < 0.05, ** *p* < 0.01, ns *p* > 0.05. The cyan cartoon indicates the PXR protein, the long stick of the red series indicate PEG400 molecules, and the short stick of the blue-colored series indicate amino acid residues of the protein, the yellow dashed line indicates hydrogen bonding.

Our study identified changes in over 40 metabolic enzymes, including phase I and phase II enzymes, forming the molecular basis for PEG400’s effects on liver metabolism, as revealed by PPI network analysis. Phase I enzymes, such as CYP, mediate the oxidative metabolism of drugs, environmental compounds, and endogenous substances, accounting for the metabolism of over 60% of drugs. PEG400’s effects on CYP activity may pose a risk of drug–drug interactions. Phase II enzymes, such as UDP-glucuronosyltransferases (UGTs), catalyze glucuronidation reactions, altering substrate activity and influencing drug pharmacokinetics. Different isoforms of UGT enzymes differ in their substrate specificity. For example, Ugt2b7 specifically metabolizes morphine to produce morphine-6-glucuronate (M6G) and morphine-3-glucuronate (M3G), of which M6G has a higher strong analgesic activity [22]. Our results showed that PEG400 upregulated Ugt1a9 while downregulating Ugt2b, Ugt2b7, and Ugt2b15. Our previous studies have demonstrated that PEG400 enhances the bioavailability and anti-inflammatory activity of baicalin by inducing Ugt1a8 and Ugt1a9 [10,11]. We have recently published a study showing that PEG400 affects the pharmacokinetics of mycophenolic acid by inducing the activity of metabolic enzymes and inhibiting efflux transporters, and Ugt1a9 is one of the major biphasic metabolic enzymes for mycophenolic acid metabolism in vivo [23]. The nuclear receptor family regulates the expression of metabolic enzymes. We used WB to detect the pregnane X receptor (PXR) and its regulated CYP enzymes. We found that PXR was activated, the expression of Cyp2a6 was downregulated, and the changes in Cyp3a4 were not significant (Figure 8A). We examined the interaction between PEG400 and PXR by molecular docking. We found that PEG400 could interact with serine and glutamine amino acid residues of PXR by hydrogen bonding with a binding energy of −5.1 KJ/mol (Figure 8B). This suggests that PEG400 may induce nuclear receptor activation. It has been known previously that PEG400 induces activation of the PPAR signaling pathway, suggesting that PEG400 may induce nuclear receptor activation, thereby regulating the expression of metabolic enzymes.

## 3. Discussion

Pharmaceutical excipient PEG400 is widely used in drug formulations due to its excellent biocompatibility and amphiphilic nature. Its content can exceed 50% in certain formulations, ensuring the proper release of active pharmaceutical ingredients and enhancing drug bioavailability. Increasing evidence suggests that PEG400 affects the activity of drug-metabolizing enzymes and transporters, thereby altering drug pharmacokinetics. The liver, the primary site of drug metabolism and biotransformation, contains numerous drug-metabolizing enzymes. In this study, we utilized proteomics to investigate the impact of PEG400 on rat liver metabolism. We observed significant changes in the expression of multiple metabolic enzymes, offering insights into the potential mechanisms of excipient–drug interactions.

Our TMT-labeled quantitative proteomics analysis revealed significant differences in the liver proteome between the control and PEG400 groups. Compared to the control group, 64 proteins were upregulated and 89 proteins were downregulated in the PEG400 group. These changes involved several biological processes that could directly or indirectly influence the metabolism of endogenous substances and physiological functions in rats. Further analysis indicated that PEG400 mainly affected pathways including retinol metabolism, bile acid synthesis and transport, and the PPARα signaling pathway, which regulates lipid metabolism, fatty acid oxidation, and transport.

Retinol (vitamin A) is one of the 13 essential vitamins required for vision and skeletal growth. In the liver, retinol binds to retinol-binding protein 1 and undergoes oxidation by NAD^+^-dependent retinol dehydrogenase to form retinaldehyde, which Aldh1a1 then catalyzes to produce RA [24]. RA is a lipid-soluble signaling molecule that regulates cell differentiation, proliferation, and apoptosis. RA and 9-cis-RA are natural ligands for the nuclear receptors RAR and RXR, and the biological activity of RA is mainly achieved by binding to the corresponding nuclear receptors [25]. Our results demonstrated that PEG400 promotes retinol metabolism to RA by upregulating Aldh1a1 and Cyp4a11 expression. Aldh1a1 is an enzyme involved in RA biosynthesis and REDOX balance, detoxifying harmful aldehydes and promoting RA production [26]. CYPs and Ugt2b7 are involved in the metabolic transformation of RA. The accumulated RA in the liver binds specifically to RAR or RXR to form dimeric complexes and then plays a series of physiological functions. This process modulates cell proliferation, differentiation, apoptosis, regulates gene expression, and so on [27,28,29]. RXR activated by RA can also form heterodimers with other nuclear receptors such as the farnesoid X receptor (FXR), PXR, and PPARα, thereby regulating the expression of downstream target genes [30]. In this study, we found that the protein expression of the nuclear receptor RXRα was increased, which may be related to the activation of RXRα by PEG400, which promotes the production of RA, the ligand of RXR.

Bile acids are amphipathic steroid molecules derived from hepatocyte cholesterol catabolism, through CYP enzyme-mediated oxidation [31]. Primary bile acids synthesized in the liver are conjugated with taurine or glycine, stored in the gallbladder, and secreted into the duodenum, where they are reabsorbed in the ileum or excreted. Amacr is considered to be an indispensable step in the synthesis of bile acids, responsible for catalyzing the isomerization of bile acid intermediates [32]. In the liver, bile acid synthesis involves enzymes such as bile acid-CoA synthetase (BACS) and BAAT. Our study revealed significant upregulation of Amacr and BAAT expression in the PEG400 group, resulting in increased bile acid content in the liver and small intestine. Bile acids are considered endogenous ligands of a series of nuclear receptors, including FXR, PXR, PPAR, constitutive androstane receptor (CAR), etc. [33]. Bile acids, as natural ligands of FXR, can activate FXR to play a role as a signaling molecule. FXR activation promotes hepatic fatty acid β-oxidation, enhancing lipid breakdown for energy while suppressing lipid synthesis, thus mobilizing fat metabolism [34]. Studies have shown that intestinal barrier damage in IBD patients and mice with colitis is related to the activation of FXR and PPARα by dysregulation of bile acid metabolism [35]. Bile acids can also induce the expression of PPARα and promote fatty acid oxidation in the liver by activating FXR [36].

The cytochrome oxidase Cyp3A is a downstream target of FXR and PXR regulation and plays an important role in the detoxification of bile acids [37,38]. In the present study, we did not detect changes in CAR and FXR, but the protein expression of both PXR and PPARα was upregulated, indicating that these two nuclear receptors were activated. We also examined the protein expression of Cyp2a6 and Cyp3a4, which are closely related to drug metabolism; Cyp2a6 was downregulated, while Cyp3a4 was not significantly altered. Previous studies have reported that PEG400 can activate CYP3A4 expression, which may be related to the activation of nuclear receptors by PEG400 promoting bile secretion.

The PPAR signaling pathway plays a key role in regulating fatty acid degradation, bile acid biosynthesis, phospholipid metabolism, and lipid metabolism [39]. Our study observed significant activation of the PPARα signaling pathway and changes in PPARα-regulated target proteins associated with lipid metabolism. PPARα activation inhibits adipocyte formation and differentiation while promoting fatty acid β-oxidation and degradation, which may explain the slower body weight gain in the PEG400 group. Previous studies have shown that oral administration of 40% PEG400 reduces obesity in high-fat diet-fed mice [7]. Our previous study also found that feeding mice with high doses of PEG400 caused weight loss in mice [8]. Consistent with this, our findings suggest that the PPARα signaling pathway may be involved in PEG400-mediated anti-obesity effects. Furthermore, PPARα regulates the expression of metabolic enzymes and transporters. For example, PPARα promotes the expression of Cyp4a genes, which catalyze the hydroxylation of unsaturated fatty acids at ω- and ω1-positions, accelerating fatty acid oxidation [39]. This may explain the significant upregulation of Cyp4a2, Cyp4a11, and Cyp4a12 expression observed in the PEG400 group. The effects of PEG400 on PPARα signaling may explain its role in mitigating obesity.

The regulation of metabolic enzyme expression involves multiple nuclear receptors, including CAR, PXR, FXR, and PPAR [40,41,42,43]. For example, FXR regulates Ugt2b4 expression, while PXR controls Ugt2b7 and Ugt1a expression [44]. Studies have reported that compounds such as Byakangelicin activate CYP3A4 expression via PXR in human hepatocytes [45]. Our WB results showed that PEG400 significantly upregulated the protein expression of Ugt1a9 and Cyp4a11 and significantly downregulated the protein expression of Ugt2b7. These effects may be related to PEG400-induced nuclear receptor activation. Our research suggests that PEG400 promotes the generation of RA and the synthesis and transport of bile acids, both of which are endogenous ligands of nuclear receptors and can bind to them to promote their activation. In addition, RXR can form heterodimers with PXR, FXR, and PPARα, thereby regulating the expression of metabolic enzymes and transporters [21,24,25,26]. Indeed, increased protein expression of RXRα, PPARα, and PXR was observed in the present study. Molecular docking results suggest that PEG400 itself may also directly interact with PPARα or PXR, activating PPARα or PXR to form heterodimers with RXRα and further regulating downstream target proteins. These interconnected processes collectively regulate hepatic metabolism, indicating that PEG400’s effects on these biological pathways are interrelated.

**Figure 9:**
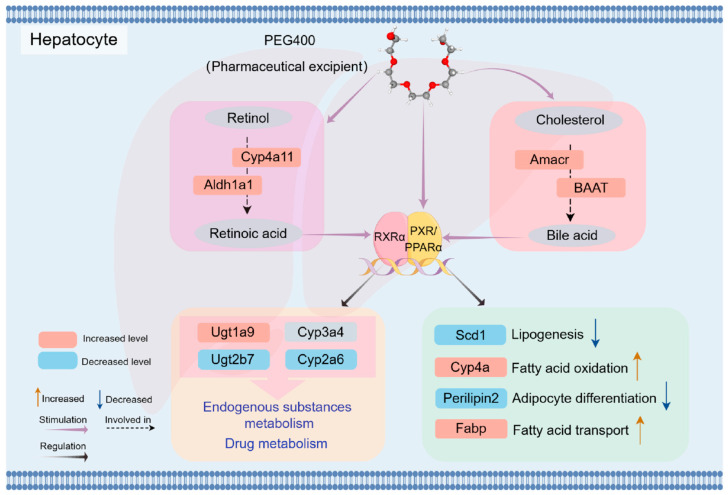
Schematic representation of the possible effects of PEG400 on rat liver metabolism. Gray ovals represent metabolites, and squares represent related enzymes and regulatory proteins. Pink indicates increased levels, and blue indicates decreased levels. Purple arrows indicate stimulation, black arrows indicate regulation, and black dashed arrows indicate involvement.

In the final analysis, PEG400 significantly changed the expression of proteins in the liver of rats, promoted the metabolism of retinol to produce retinoic acid, and enhanced bile acid synthesis and secretion. Additionally, RA and bile acids, as natural ligands of nuclear receptors, activate nuclear receptor pathways to regulate metabolic enzymes and transporters. Figure 9 shows a schematic representation of the possible effects of PEG400 on rat liver metabolism. Firstly, PEG400 promotes the metabolism of retinol to produce retinoic acid and increases the synthesis and secretion of bile acids. As endogenous ligands of nuclear receptors, RA and bile acids activate nuclear receptors such as PPAR and PXR. Alternatively, PEG400 may act as a lipid-soluble molecule and directly activate the expression of nuclear receptors, thereby affecting the expression of lipid and metabolic enzymes. These findings suggest that PEG400, beyond its traditional physical role as an excipient, may affect the expression of metabolic enzymes by inducing nuclear receptor activation, thereby influencing endogenous metabolic pathways and drug pharmacokinetics. This study provides a comprehensive understanding of the effects of PEG400 on rat liver metabolism, offering new insights for its application in drug formulations and supporting the development of novel clinical therapies and formulations.

## 4. Materials and Methods

### 4.1. Reagents and Materials

PEG400 was purchased from Beijing Solarbio Science & Technology Co., Ltd. (Beijing, China). Protease Inhibitor Cocktail was purchased from Merck Millipore (Burlington, MA, USA). Trypsin, Urea, Dithiothreitol, iodoacetamide, and tetraethyl ammonium bromide (TEAB), supplied by Sigma-Aldrich (St. Louis, MO, USA), were used for proteomic analysis. The Tandem mas Label (TMT) kit was provided by Thermo Fisher Scientific (Waltham, MA, USA), except UPLC-grade reagents (formic acid, acetonitrile, and methanol), which were purchased from Merck (Darmstadt, Germany). The rat retinoic acid (RA) ELISA kit was obtained from FANKEW (Shanghai, China). Total bile acid (TBA) assay kits were purchased from Nanjing Jiancheng Technology Co., Ltd. (Nanjing, China). Abmart (Berkeley Heights, NJ, USA) supplied the primary antibody for Ugt1a9. Baijia delivered antibodies specific to Amacr, BAAT, PPARα, RXRα, PXR, Fabp1/5, Cyp3a4, and Cyp2a6. Huabiotech (Shanghai, China) supplied Scd1, Perilipin2, Cyp4a11, and GAPDH antibodies. Proteintech (Rosemont, IL, USA) provided Ugt2b7 antibody. β-actin antibody was derived from Servicebio (Wuhan, China). All other reagents and chemicals were of analytical grade.

### 4.2. Animal Experiments

Male Sprague–Dawley rats (230 ± 10 g) were obtained from Changsha Tianqin Biotechnology Co., Ltd., (Changsha, China) (Permit Number: SCXK [Xiang] 2019-0014). Six healthy male rats were divided into two groups (normal group and PEG400 group) by average weight, and they were housed for seven days at a temperature of 24 ± 2 °C, a humidity of 60 ± 5%, with a 12 h light/dark cycle, and a free diet before the experiment. The PEG400 group was given intragastric PEG400 (10 mL/kg), and the normal group was given the same amount of saline. The administration continued for seven days, and the diet was normal during the experiment period. The body weight and living conditions of the rats were monitored daily. After seven days, rats were sacrificed, and then biological samples were collected and rapidly stored at −80 °C. All animal studies were approved by the Animal Care Welfare Committee of Guizhou Medical University (Permit Number: SYXK[GUI]2023-2002) and strictly followed the operating procedures of animal experiments.

### 4.3. Liver Histopathology

The liver tissues of rats were preserved in 4% paraformaldehyde, dehydrated with various concentrations of ethanol, cleared with xylene, embedded in paraffin, cut into 4 µm slices, and dyed with hematoxylin and eosin (HE). Afterward, the sections were examined and photographed using a light microscope.

### 4.4. TMT Labeling Quantitative Proteomics Analysis

In this section, a series of technologies such as protein extraction, enzyme digestion, TMT labeling, high-performance liquid chromatography (HPLC) classification, liquid chromatography–mass spectrometry tandem analysis, database search, and biological information analysis were used to study the quantitative proteome of samples [46]. Briefly, appropriate amounts of hepatic tissue were ground into a powder using liquid nitrogen. The resulting powder was sonicated on ice with 4 volumes of lysis buffer (8 M urea, 1% protease inhibitor). After centrifugation at 12,000× *g* for 10 min at 4 °C, the protein concentration of the supernatant was determined using a BCA kit (Solarbio). Next, the protein solution was digested with trypsin, desalted using a Strata X C18 solid-phase extraction column (Phenomenex, Torrance, CA, USA), and vacuum-dried to obtain peptides. The peptides were then labeled with TMT according to the kit’s instructions. Subsequently, the TMT-labeled peptides were separated by high pH RP-HPLC using an Agilent 300 Extend C18 column (4.6 × 250 mm^2^, 2.5 μm, Agilent, Santa Clara, CA, USA). The separated peptides were detected using an Orbitrap Exploris 480 nanoelectrospray (Thermo Fisher Scientific, Waltham, MA, USA) ion source, resulting in mass spectrometry data. Mass spectrometry data were retrieved using Maxquant (v1.6.15.0). Finally, the raw data were compiled and analyzed to identify differentially expressed proteins. Proteins with fold changes >1.2 or <1/1.2 and *p* < 0.05 were considered significantly expressed proteins. The differentially expressed proteins (DEPs) were further analyzed using bioinformatics tools such as GO, KEGG, and PPI.

### 4.5. Determination of Retinoic Acid and Bile Acid

Serum and liver tissues were collected for retinoic acid detection. Serum, liver, and ileum tissues were obtained for bile acid detection. Retinoic acid and bile acid were determined using assay kits following the manufacturer’s instructions.

### 4.6. Molecular Docking

The utilization of molecular docking simulation represents a convenient and efficacious approach for investigating the interaction between molecules and protein targets. The crystal structure of the candidate protein was obtained from the RCSB protein database. These structures underwent modifications using the AutoDock Tool 1.5.7 software, including hydrogenation, dehydration, ligand removal, and amino acid optimization. The modified structures were saved in pdbqt format. The three-dimensional chemical structures of PEG400 were downloaded from PubChem, subjected to energy minimization, and saved in MOL.2 format. The compound was subsequently imported into AutoDock Tool 1.5.7, where all flexible bonds were configured to be rotatable by default and saved as docking ligands in pdbqt format. Docking studies were performed using AutoDock Vina 1.1.2, and the results were visualized using PyMOL 2.5.

### 4.7. Western Blotting Verification

The significantly altered metabolic pathways were gained through proteome analysis. The differential proteins in these pathways were verified by WB experiments to obtain specific experimental data on the effect of PEG400 on liver metabolism. The liver tissue was used for total protein extraction following the RIPA lysis buffer protocol provided by NCM Biotech, Newport, RI, USA. Protein concentrations were quantified using the BCA kit. The proteins were subsequently separated on 10% SDS-PAGE gels and transferred to PVDF membranes from Millipore. The membrane was blocked with 5% skim milk for 2 h at room temperature and then incubated overnight at 4 °C with the primary antibody. The dilution ratios of primary antibodies are shown in Table 1. After three washes with TBST, the membrane was incubated with appropriate secondary antibodies (1:5000) for 2.0 h at room temperature then washed with TBST. The protein signal was visualized using a gel imaging system (Shanghai Tianneng, Shanghai, China) and an ultra-sensitive ECL detection kit from Biosharp. The intensity of the bands was measured using ImageJ software 1.8.0, and the data were visualized using GraphPad Prism 8 software.

### 4.8. Statistical Analysis

The Fisher exact test was used for enrichment analysis and *p*-value assessment of differential expression in proteomic analysis. The statistical analysis between the two groups was performed using a Student’s unpaired *t*-test in SPSS 23.0. Results are expressed as the mean ± SEM and were obtained from three separate experimental trials. A significance level of *p* < 0.05 was considered for statistical analysis. GraphPad Prism 8 was utilized for visual analysis of the results.

## Figures and Tables

**Table 1:** Antibodies used in this study.

Antibody	Host	Western Blot Dilution	Item No.
Aldh1a1	Rabbit	1:2000	IPB0967
Cyp4a11	Rabbit	1:1000	HA722348
Ugt2b7	Rabbit	1:2000	16661-1-AP
Amacr	Mouse	1:1000	IMB0226
BAAT	Rabbit	1:2000	IPB6862
ARαPP	Rabbit	1:2000	IPB0660
RXRα	Rabbit	1:2000	IPB8970
Perilipin2	Rabbit	1:1000	ET1704-17
Scd1	Mouse	1:1000	HA601180
Fabp5	Rabbit	1:2000	IPB7383
Ugt1a9	Rabbit	1:1000	TD6537
Cyp3a4	Rabbit	1:500	IPB2432
Cyp2a6	Rabbit	1:500	IPB0396
PXR	Rabbit	1:2000	IPB0584
GAPDH	Rabbit	1:8000	ET1601-4
β-actin	Rabbit	1:5000	GB15003

## Data Availability

Data are contained within the article.
